# Diagnostic Utility of Magnetic Resonance Imaging in Eosinophilic Fasciitis: A Case Report and Review of Literature

**DOI:** 10.7759/cureus.37899

**Published:** 2023-04-20

**Authors:** Jiwon Kim, Lit Hiang Lee

**Affiliations:** 1 General Medicine, Goulburn Valley Health, Shepparton, AUS

**Keywords:** magnetic resonance imaging, connective tissue disease, shulman syndrome, eosinophilia, eosinophilic fasciitis

## Abstract

Eosinophilic fasciitis (EF), also known as Shulman syndrome, is a rare scleroderma-like disorder that is characterized by an acute onset of induration, swelling, erythema, and tenderness of the skin and deep fascia, often affecting all four limbs. We report a case of eosinophilic fasciitis in a 51-year-old female patient, whose diagnosis of EF was made based on the findings from clinical evaluation and magnetic resonance imaging (MRI) but without skin biopsy. She was treated with a combination therapy of prednisolone and methotrexate, and her response to therapy was assessed via clinical assessment and MRI. MRI may be a useful non-invasive diagnostic tool for not only supporting but also confirming the clinical diagnosis of EF when a skin-to-muscle biopsy is not available or cannot be performed, as well as for monitoring disease activity and response to therapy. Further prospective studies should be conducted to evaluate the precise sensitivity and specificity of MRI in diagnosing EF and also to create more structured protocols to guide the diagnosis and management of EF.

## Introduction

Eosinophilic fasciitis (EF) is a rare connective tissue disorder with poorly understood etiology and pathogenesis, first described by Shulman in 1974 [1]. EF is characterized by inflammation and thickening of the skin and deeper muscle fascia, and presents clinically as rapidly progressive symmetrical induration, swelling, erythema, and tenderness of the skin and deep fascia, most commonly affecting all four limbs [2]. These clinical features are often accompanied by peripheral eosinophilia, polyclonal hypergammaglobulinemia, and elevated inflammatory markers on laboratory tests [1,3]. The widely accepted gold standard investigation to confirm the clinical diagnosis of EF is full-thickness skin-to-muscle biopsy, with imaging modalities such as magnetic resonance imaging (MRI) becoming increasingly recognized as useful adjuncts in assisting with diagnosis [4,5]. In this case report, we present a case of EF diagnosed based on clinical assessment and MRI and treated with a combination therapy consisting of prednisolone and methotrexate.

## Case presentation

A 51-year-old female patient presented with an erythematous rash on her upper back, myalgia, arthralgia, and diffuse swelling of the forearms and lower limbs. This was followed by rapidly progressive tightening and stiffness of the skin in all four limbs, but there was no significant joint swelling. Apart from her musculoskeletal symptoms, she was also experiencing significant fatigue, especially at the onset of her illness.

**Figure 1:**
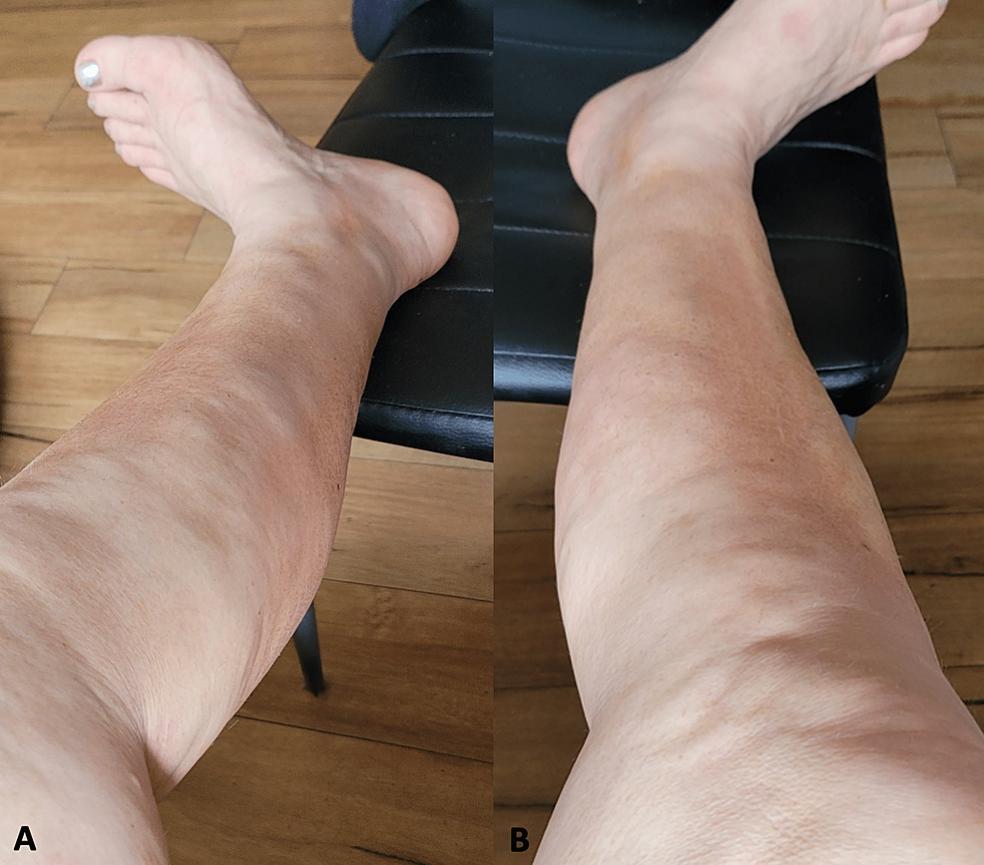
“Peau d’orange” sign on (A) left leg and (B) right leg

On examination, she had striking bilateral non-pitting edema in both her forearms and calves. The patient also gradually developed a symmetrical "woody" induration of forearms and legs with peau d’orange appearance over two to three months (Figure 1). This was associated with the emergence of a groove sign with linear depression along the superficial veins, which became most prominent at approximately five months from the onset of her initial symptoms. However, there were no signs of Raynaud’s syndrome, sclerodactyly, joint tenderness, or synovitis.

Laboratory tests revealed peripheral eosinophilia of 2.7 x 10^9^/L (normal range: 0.0-0.5) as well as elevated C-reactive protein (CRP) and erythrocyte sedimentation rate (ESR), measuring 57 (normal range: <5.0) and 25 (normal range: 1-19), respectively. The creatine kinase (CK) level was normal.

**Figure 2:**
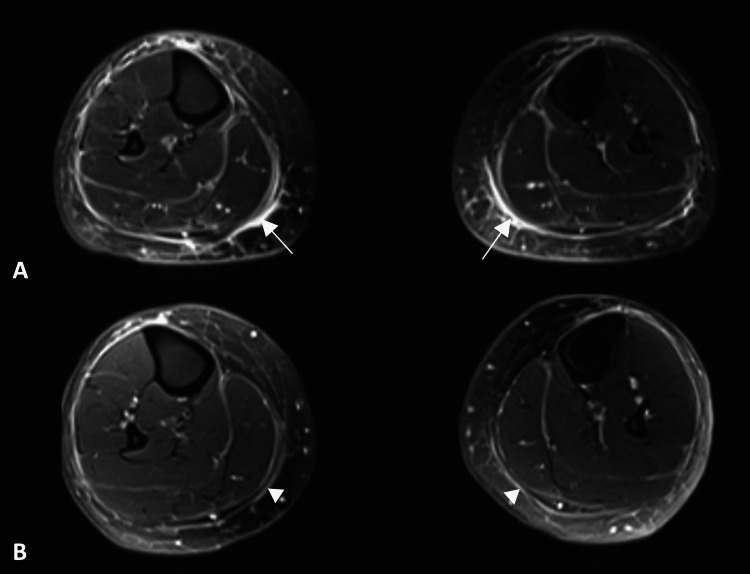
(A) Axial T2-weighted fat-suppressed MRI image demonstrating thickening of deep peripheral fasciae (arrows) of both lower legs, visualized as high signal intensity in the affected areas. (B) A repeat MRI was performed after three months, showing a clear reduction in the fascial thickening in the same region (arrowheads).

Multiplanar, multisequence MRI of the lower limbs was taken using a 3.0 Tesla scanner, which showed typical features of EF, with marked inflammation of the superficial and deep fascial layers of both legs down to the ankles (Figure 2, Panel A). The MRI changes were highly symmetrical, and there was a characteristic absence of myositis. The patient was also booked for a deep skin biopsy for a definitive diagnosis but was not able to undergo the procedure as she had incidentally caught COVID-19 infection just prior to the date of the biopsy.

The patient was commenced on prednisolone 25 mg daily. There was a prompt improvement in pain and stiffness, but she continued to experience a sensation of skin tightness. As a steroid-sparing agent, we added methotrexate therapy at 10 mg once weekly for two weeks, followed by 20 mg once weekly, along with folic acid.

Given her classic clinical features of EF, laboratory results, and characteristic MRI findings, the patient was diagnosed with EF and continued the treatment. Her blood tests showed a significant decline in CRP and eosinophil count following treatment. The addition of methotrexate gave her a noticeable improvement in her symptoms of skin and joint tightness. However, she subsequently started experiencing more symptoms of calf tightness again. Her methotrexate dose was increased to 25 mg/week, and she was also given pulsed IV methylprednisolone 1 g daily for three days. A follow-up MRI was performed following this, which showed a marked reduction of fascial thickening in the superficial and deep fascial layers of the affected areas (Figure 2, Panel B).

On continued follow-up, the patient was experiencing fluctuating tightness and burning pain in her lower legs. This was accompanied by a concurrent rise in the CRP to 17.7. Prednisolone was increased to 37.5 mg once daily, although this had to be reduced back to 25 mg daily in only three days due to gastrointestinal side effects and hot flushes. On repeat follow-up, her CRP settled to <10, and a plan was made for prednisolone therapy to be gradually weaned, guided by her symptoms and inflammatory markers.

## Discussion

EF is a rare fibrosing disorder, with a paucity of guidelines, and as a result, management is largely based on expert opinion and published case reports [2]. The etiology and pathophysiology of EF remain poorly understood, and a majority of cases are considered idiopathic [6,7]. However, there have been suggestions that EF may be occasionally associated with certain risk factors such as strenuous physical activity, autoimmune conditions, and exposure to statins [1,2,8,9].

EF most commonly presents with cutaneous involvement as reported in the vast majority of patients [2,9,10]. Symptoms often evolve temporally, beginning with an edematous phase, followed by skin induration with a "peau d’orange" appearance, which then progresses to tightness and stiffness of the skin and deep fascia [2,11,12]. Cutaneous symptoms typically affect all four limbs in symmetry, but they can sometimes occur unilaterally and involve the trunk. Onset is often acute, although subacute courses have also been described [2]. Myalgia is another commonly reported symptom of EF, affecting up to 86% of patients [2,11]. Visceral involvement of the lungs, heart, and kidneys has also been reported, but this is rare and has only been reported in a few case reports [9,13-15]. Joint contractures can occur as a result of fibrosis of the subcutaneous tissue from disease progression [16].

Laboratory findings in patients with EF are variable, with the most common finding being peripheral eosinophilia, seen in approximately 60%-90% of cases [2,5,17]. Other common findings include polyclonal hypergammaglobulinemia and elevated inflammatory markers, both of which are seen in more than 50% of patients [2]. While peripheral eosinophilia is a characteristic feature of EF, it is not required for diagnosis and is a transient finding that does not correlate with disease severity, with levels fluctuating over time independent of the disease activity [4,9,10,17]. However, there have been reports hypothesizing a potential correlation between disease activity in EF and other biomarkers such as serum aldolase levels [18]. Serum CK levels are usually normal, even in the presence of myalgia, as observed in our patient [9,19].

The widely accepted gold standard investigation to establish a definitive diagnosis of EF is a full-thickness skin-to-muscle biopsy, extending from the epidermis to the muscle [1,5,7,9,20-23]. The possible findings from a histopathological examination of the biopsy in EF include marked fascial thickening, edema of the fascia, and deep subcutis with inflammatory infiltrates such as eosinophils [20,23,24]. However, a diagnostic challenge is posed when a deep skin biopsy cannot be obtained, as was the case for our patient. We believe that in such situations, MRI can be an important tool for the diagnosis of EF when histopathological confirmation is unattainable.

The utility of MRI in the diagnosis and monitoring of EF has been evaluated in multiple reports, which we identified and explored through a literature search on MEDLINE, Embase, and PubMed databases. The main MRI finding in EF patients is the thickening of muscle fasciae with high signal intensity or contrast enhancement of the fascia, most often sparing the adjacent musculature and subcutaneous adipose tissue [4,5,7,23,25,26]. This finding is best demonstrated on fat-saturated spin-echo sequences that eliminate phase artifacts, which may arise as a result of the anatomical proximity between fascia and hypodermis [7,21]. Such sequences include T1-weighted sequence with gadolinium contrast, fat-suppressed T2-weighted sequence, and short tau inversion recovery (STIR) sequence [4,7,21]. Ronneberger et al. reported cases of two patients whose diagnosis of EF was made on the basis of MRI findings without positive skin-to-muscle biopsy results [27]. Sugimoto et al. also reported a case where EF was diagnosed largely on clinical evaluation and MRI findings without biopsy [28]. Furthermore, a Japanese guideline developed by Jinnin et al. in 2017 suggested diagnostic criteria that recognize the utility of MRI in establishing the diagnosis of EF without the need for a histological examination, provided that the patient displays typical clinical features of EF [29].

MRI also plays a significant role in monitoring the disease activity, determining the effectiveness of therapy, and confirming the remission of the disease [4,21,23,26,28,30]. The extent of MRI signal abnormalities has been shown to parallel disease activity, and as the disease resolves, the findings on MRI also resolve, and signs of fascial inflammation become absent, as evident in our case [23,25,28]. This is especially more useful, given the lack of a well-established laboratory marker that correlates with disease activity. Moulton et al. described three cases of EF, where MRI demonstrated a definite improvement after corticosteroid therapy [25]. Sugimoto et al. also reported a case where a follow-up MRI scan post-therapy demonstrated a considerable reduction in the fascial signal intensity of the area affected by EF [28]. Thus, findings from MRI can be used to monitor the course of the disease, identify whether patients have responded to therapy, subsequently guide the duration of therapy, or suggest the need for stronger therapy in refractory cases [4,5].

Although not explored in our case report, MRI is also ideal for selecting the optimal site for biopsy as it clearly highlights the areas of inflamed fascia [4,25,26]. Baumann et al. described six cases where the sites of biopsies were all guided by MRI and yielded positive results [4]. Furthermore, Grados et al. reported the cases of three patients with EF who all had negative first muscle biopsies and later had positive biopsies with the aid of MRI in selecting the site for biopsy [31]. Hence, MRI can be considered a useful non-invasive tool in minimizing the chances of obtaining false-negative results from skin-to-muscle biopsies and the need for repeat procedures.

There is currently no randomized controlled trial or internationally agreed consensus to guide the treatment for EF, and its management is based on anecdotal reports and reviews of the existing literature. The generally agreed first-line therapy for EF among published literature is corticosteroid therapy, with more than 70% of patients showing either a partial or complete response to treatment [2,9,19]. There is no well-defined recommendation for the duration of corticosteroid therapy in treating EF, and the doses are usually tapered according to how well the patients respond to therapy [2,10]. While corticosteroids are the mainstay of therapy, a range of immunosuppressive/immunomodulatory drugs have also been used in the treatment of EF, including methotrexate, hydroxychloroquine, mycophenolate, cyclosporine, rituximab, and immunoglobulins [9,12,19,32,33].

## Conclusions

We report a case of EF where the diagnosis was made based on clinical evaluation and MRI findings without skin-to-muscle biopsy. Our case report illustrates that MRI can be a useful diagnostic tool for not only supporting but also confirming the clinical diagnosis of EF where skin-to-muscle biopsy is not available or cannot be performed. MRI is non-invasive and offers the additional advantage of providing an objective assessment of response to therapy. Hence, in cases where clinical and laboratory features are classic for EF, MRI is a valuable alternative to a skin-to-muscle biopsy. Further prospective studies should be conducted to help evaluate the sensitivity and specificity of MRI in the diagnosis and monitoring of EF.
